# Infantile Paralysis or Acute Anterior Poliomyelitis

**Published:** 1916-09-09

**Authors:** 


### INFANTILE PARALYSIS OR ACUTE ANTERIOR POLIOMYELITIS.

### Its Symptoms and Treatment.

This mysterious disease seldom occurs in epidemics in this country, though individual scattered cases are not very rare, and must amount to a goodly total in the British Isles each year. In certain foreign countries, however, notably in America and in Scandinavia, it now and then blazes up into serious epidemics. The most serious outburst recorded for many years is at present afflicting New York, and to a lesser extent the neighbouring towns in New Jersey and Connecticut. Over 6,000 cases have been reported during the two months ending August 14, with the appalling total of 1,500 deaths. Not only is this the largest epidemic of recent times, but the death-rate is also exceptionally high. Experience shows that August and September are the two months when poliomyelitis is most to be dreaded, so it is quite probable that before this epidemic abates it may have claimed a tale of victims even more disastrous than that already registered. And there is, unfortunately, ample time yet before the summer season closes for the complaint to make its appearance in Great Britain. Owing to the fact that the natural history of the disease is as yet undiscovered, its micro-organism unisolated, and the method of spread of the contagion quite uncertain, there is great difficulty in devising measures for preventing the importation of the infection; and a ten-days' quarantine for all arrivals from the infected parts of the United States has been suggested as desirable.

### The Clinical Picture.

Infantile paralysis was first described by Underwood, an English physician, in 1774, though it was not until nearly a hundred years later that really full and careful accounts of it were available. It has been suggested that Mephibosheth, whose paralysis is attributed in Scripture to a fall, was in reality a sufferer from anterior poliomyelitis. All sorts of causes have been ascribed for its appearance, probably without much foundation: injuries to the back, over-exertion, teething, sitting in a draught or on damp grass are some of these. It seems to be established that the early symptoms are nearly always those of a slight sore throat, with feverishness, vomiting, malaise. After two or three days—sometimes even a week—it is suddenly discovered that the patient is paralysed somewhere; usually in one or more limbs, but sometimes in the diaphragm as well, indeed universally. At times this leads to a provisional diagnosis of diphtheria; but diphtheria bacilli are not found on the fauces. Headache, convulsions, incessant vomiting, and diaphragmatic palsy are symptoms of the severe cases which often end fatally. More commonly paralysis is limited to one limb, and even there may be but partial. The leg is more often affected than the arm, and the calf muscles more often than those of the thigh. After a week or two a gradual improvement sets in as a rule, and this may progress to complete recovery. But very often there remains some degree of permanent paralysis of the affected part. In the present New York epidemic it is stated that the percentage of the permanently crippled is high; this is what might be expected along with the high death-rate, for evidently the infection is of a very virulent type. Greater or less degrees of permanent deformity are not uncommon as a result of poliomyelitis. Sir Walter Scott was permanently lame through an attack of this disease in childhood.

Children are the chief sufferers. Ninety per cent. of the cases occur in the first five years of life, and most of these are under two and a half. But older children and adults are not immune, and several of the latter have perished recently in New York. American investigators have succeeded in infecting monkeys with the disease, and have shown that the virus can pass through a Berkefeld filter; it must therefore be more minute than any of the known micro-organisms. In fatal cases inflammation, hæmorrhages, and softening have been found in the anterior horns of the grey matter of the spinal cord, to damage of which the symptoms of paralysis are undoubtedly due. In some cases the infection attacks also, or chiefly, the grey matter of the brain (acute polioencephalitis), in which cases death is very common though not invariable.

### Treatment.

Treatment of the acute febrile stage is mainly symptomatic; indeed, the nature of the trouble is seldom diagnosed at first until paralysis appears. Rest in bed, with fever diet and necessary aperients, is the basic principle. The limbs must be kept warm and dry; if there is pain in them, belladonna and glycerine will be found a useful application. Dry hot cotton wool, or Thermogene wool, may be used to enwrap the affected limbs. No drugs have any influence in this stage of the trouble.

As soon as the acute symptoms have subsided and the temperature becomes normal, local treatment of all paralysed parts must be commenced without any delay. Electrical stimulation of the paralysed muscles, combined with massage, is most important. Passive movements of joints, a cradle to prevent foot-drop, and careful precautions against all possible contractures and deformities are to be strictly observed. General tonic treatment is also of much value, especially an open-air régime. Syrup of iron, strychnine, maltine, phosphates and hypophosphites, and cod-liver oil are all of value, singly or in combination. Exercises, suitably controlled, come in useful later on in restoring function to wasted or semi-paralysed limbs. After months have passed by surgery may be needed to correct deformities, but never in recent cases, as much improvement often occurs in apparently hopelessly damaged limbs.

